# Understanding the Physiopathology Behind Axial and Radial Diffusivity Changes—What Do We Know?

**DOI:** 10.3389/fneur.2018.00092

**Published:** 2018-02-27

**Authors:** Pawel J. Winklewski, Agnieszka Sabisz, Patrycja Naumczyk, Krzysztof Jodzio, Edyta Szurowska, Arkadiusz Szarmach

**Affiliations:** ^1^Department of Human Physiology, Medical University of Gdańsk, Gdańsk, Poland; ^2^Department of Clinical Anatomy and Physiology, Pomeranian University in Słupsk, Słupsk, Poland; ^3^2-nd Department of Radiology, Medical University of Gdańsk, Gdańsk, Poland; ^4^Institute of Psychology, University of Gdańsk, Gdańsk, Poland

**Keywords:** diffusion tensor imaging, axial diffusivity, radial diffusivity, myelin dysfunction, axonal injury

## Abstract

The use of the diffusion tensor imaging (DTI) is rapidly growing in the neuroimaging field. Nevertheless, rigorously performed quantitative validation of DTI pathologic metrics remains very limited owing to the difficulty in co-registering quantitative histology findings with magnetic resonance imaging. The aim of this review is to summarize the existing state-of-the-art knowledge with respect to axial (λ_║_) and radial (λ_┴_) diffusivity as DTI markers of axonal and myelin damage, respectively. First, we provide technical background for DTI and briefly discuss the specific organization of white matter in bundles of axonal fibers running in parallel; this is the natural target for imaging based on diffusion anisotropy. Second, we discuss the four seminal studies that paved the way for considering axial (λ_║_) and radial (λ_┴_) diffusivity as potential *in vivo* surrogate markers of axonal and myelin damage, respectively. Then, we present difficulties in interpreting axial (λ_║_) and radial (λ_┴_) diffusivity in clinical conditions associated with inflammation, edema, and white matter fiber crossing. Finally, future directions are highlighted. In summary, DTI can reveal strategic information with respect to white matter tracts, disconnection mechanisms, and related symptoms. Axial (λ_║_) and radial (λ_┴_) diffusivity seem to provide quite consistent information in healthy subjects, and in pathological conditions with limited edema and inflammatory changes. DTI remains one of the most promising non-invasive diagnostic tools in medicine.

## Introduction

The number of studies using diffusion tensor imaging (DTI) has grown exponentially. A search for the term “diffusion tensor imaging” yields 13,841 records in PubMed. This is not surprising as DTI represent a highly promising method for characterizing microstructural evolution in neuropathology and treatment (1).

For instance, DTI allows developmental changes in the prefrontal cortex to be tracked. It is believed that brain maturation is associated with augmented myelination, organization, and integrity of frontal white matter; this is confirmed by DTI indices, such as fractional anisotropy, mean diffusivity, radial diffusivity (λ_┴_), and axial diffusivity (λ_║_). Therefore, DTI provides a tool to highlight patterns associated with the developmental time course of the frontal structural integrity, which correlates with the improvements in higher level cognitive functions taking place between adolescence and early adulthood (2). Interestingly, DTI studies reveal some consistent patterns in subjects exhibiting antisocial behavior. In particular, adult antisocial behavior was shown to be associated with greater diffusivity within several white matter tracts, including the inferior fronto-occipital fasciculus, uncinate fasciculus, cingulum, thalamic radiations, corticospinal tract, and corpus callosum (3).

At the same time, a clinical meta-analysis in subjects with mild-traumatic brain injury produced conflicting results. This large review, consisting of 86 studies, concluded that “DTI is sensitive to a wide range of group differences in diffusion metrics, but currently lacks the specificity necessary for meaningful clinical application” (4). There is a clear lack of consensus among the experts about the use of DTI indices in a specific region of the brain as biomarkers for post-concussion syndrome, because no consistent trends for DTI variables in these subjects have been defined (5). On the contrary, in subjects suffering from cerebral small vessel disease, associations between DTI parameters and cognition have been confirmed in a multicenter study (6).

When reviewing the DTI studies it is quite striking that rigorous quantitative validation of DTI pathologic metrics remains very limited, most likely due to the difficulty in co-registering quantitative histopathology data with magnetic resonance imaging (MRI). It seems obvious that heterogenic pathologies within the brain white matter, including changes, such as brain edema and the inflammatory response, can potentially affect the consistency of DTI metrics. The aim of this review is to summarize the existing state-of-the-art knowledge with respect to axial (λ_║_) and radial (λ_┴_) diffusivity as DTI markers of axonal and myelin damage, respectively.

## Technical Considerations

The principles of diffusion-weighted MRI were first described in the mid-1980s (7–9); they were based on the concept of MRI imaging combined with bipolar magnetic field gradient pulses that were introduced earlier to encode molecular diffusion effects on the spin-echo experiment (10). In ideal conditions, diffusion can be considered a truly three-dimensional process. However, in biological materials like tissues, molecular mobility may be constrained or facilitated in particular directions. Such anisotropy results from the presence of obstacles that influence molecular movement. The MRI signal is generated from water molecules by combining radiofrequency pulses with magnetic field gradients. Importantly, only molecular movements occurring within the direction of the gradient pulses are encoded in MRI generated signal. Consequently, the effect of diffusion anisotropy is easily measured by alternating the direction of the gradient pulses and observing variations in diffusivity parameters in the three planes. This feature makes diffusion-weighted MRI unique and distinguishes it from routine T1 or T2 weighted images (11).

Optimization of MRI sequences and fine tuning of the processing and display of recorded MRI signals allows for the full extraction of diffusion anisotropy effects, and thus, provides details on tissue microstructure. This more rigorous and elaborated diffusion-weighted MRI technique is named DTI (12, 13). The DTI matrix is obtained from a series of diffusion-weighted images in various gradient directions. The three diffusivity parameters (λ_1_, λ_2_, and λ_3_), are generated by matrix diagonalization. Diffusivities are scalar indices describing water diffusion in a specific voxels (the smallest volumetric elements in the image) associated with the geometry of white matter tracts (14, 15). The diffusivities (λ_1_, λ_2_, and λ_3_) obtained by DTI matrix diagonalization can be delimitated into parallel (λ_1_) and perpendicular (λ_2_ and λ_3_) components to the axonal tract (16–18). Axial diffusivity, λ_║_ ≡ λ_1_ > λ_2_, λ_3_, describes the mean diffusion coefficient of water molecules diffusing parallel to the tract within the voxel of interest. Similarly, radial diffusivity, λ_┴_ ≡ (λ_2_ + λ_3_)/2, can be defined as the magnitude of water diffusion perpendicular to the tract (19, 20). Fractional anisotropy in brain measures the total magnitude of water directional movement along the axonal fibers (16), while mean diffusivity is a measure of mean diffusion of each direction. Therefore, fractional anisotropy may be enhanced in situations of facilitated parallel diffusivity, restricted perpendicular diffusivity, or as a result of combination of both factors (21).

**Figure 1:**
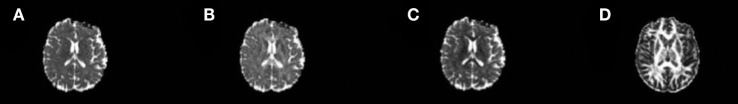
Example of maps computed from diffusion tensor imaging of the brain: **(A)** mean diffusivity, **(B)** axial diffusivity (λ_║_), **(C)** radial diffusivity (λ_┴_), and **(D)** fractional anisotropy.

White matter in the brain, organized in bundles of axonal fibers running in parallel, is the natural target for imaging based on diffusion anisotropy. In principle, diffusion along the fibers should be faster than in the perpendicular direction. Based on the assumption that the direction of the fastest diffusion indicates the overall orientation of the fibers, color-coded maps of white matter tracts in the brain are created [Figure 1 (22)]. A non-invasive method to objectively quantify white matter abnormalities greatly support studies aiming at clarification of mechanisms of damage, matching pathology with neurologic function, and assessing therapeutic interventions.

## Early Experimental Studies

White matter impairment leading to neurological disorders can be categorized according to myelin abnormality (demyelination), axonal injury, or a combination of both (23, 24). There are several animal experimental models that allow for at least partial differentiation of these processes. One such model, congenitally dysmyelinated Shiverer mutant mice, was used by Song et al. (19) in his first study on radial (λ_┴_) and axial (λ_║_) diffusivity. Radial diffusivity (λ_┴_) was significantly increased, while axial diffusivity (λ_║_) was not altered in congenitally dysmyelinated shiverer mutant mice, as compared to wild-type mice (19), suggesting that radial diffusivity (λ_┴_) may represent a potential non-invasive marker of myelin disintegration.

In a second study, Song et al. (20) used a mouse model of retinal ischemia. This model is characterized by acute inner retinal degeneration (25, 26) with initial axonal degeneration in the optic nerve, and secondary myelin fragmentation following retinal degeneration (27). Song et al. (20) observed distinct evolution patterns of axial (λ_║_) and radial (λ_┴_) diffusivity during the progression of optic nerve degeneration. Axial diffusivity (λ_║_) diminished in the injured optic nerve by day 3 following ischemia, while change in radial diffusivity (λ_┴_) between the injured optic nerves and control nerves was not detected until day 5. Radial diffusivity (λ_┴_) reached its minimal value on day 5, and remained on this level on day 7 after ischemia. Importantly, this longitudinal DTI examination of the optic nerve was positively linked with neurofilament and myelin basic protein immunostaining (28) results at day 3 (axonal degeneration) and 7 (myelin fragmentation) after the injury.

The notion that demyelination might be associated with a marked increase in radial diffusivity (λ_┴_), and modest often insignificant changes in axial diffusivity (λ_║_), was further reinforced by the third seminal study of Song et al. (29). In this study, the cuprizone model, which is characterized by consistent demyelination of the corpus callosum in mouse brains, was used. Demyelination after several weeks of diet, including cuprizone (neurotoxicant that chelates copper) is extensive, yet can be reversed if the mice are back to normal chow (30–32). The axonal’s damage time course was clearly different from the radial diffusivity time course, demonstrating that radial diffusivity (λ_┴_) recognizes demyelination as distinct from axonal damage. However, although changes in axial diffusivity measured at the initial stage of cuprizone administration suggested acute axonal damage in white matter, the diminished axial diffusivity (λ_║_) did not reach statistical significance (29). The uncertainty about the potential of axial diffusivity (λ_║_) as a marker of axon damage was further clarified in the study by Sun et al. (33), from the same group using the same cuprizone mouse model. Biweekly *in vivo* DTI examinations showed a transient decrease in axial diffusivity (λ_║_) in the corpus callosum after 2–6 weeks of cuprizone administration, while immunostaining for non-phosphorylated neurofilaments demonstrated corresponding axonal damage after 4 weeks of treatment.

In summary, in four seminal studies, Song and colleagues demonstrated that axial (λ_║_) and radial (λ_┴_) diffusivity may be useful *in vivo* surrogate markers of axonal and myelin damage, respectively, in selected mouse experimental models of white matter abnormalities.

## Critique of the Method

Interestingly, when a synthetic model of crossing fibers is used, the three diffusivities (λ_1_, λ_2_, and λ_3_) may not detect the same underlying structural characteristics in particular datasets, because orientation of the related principal eigenvector (a characteristic vector whose direction does not change in the linear transformation and has got the largest magnitude) may differ (34). According to these authors, axial (λ_║_) and radial (λ_┴_) diffusivities, i.e., the water diffusion coefficient parallel and perpendicular to the axons, may provide an acceptable approximation if the voxel includes a healthy fiber bundle determining the diffusion characteristic of the voxel. However, if the signal-to-noise ratio is low, if crossing fibers are present, or if pathology causes a decrease in anisotropy, such an approach can lead to misinterpretation of the results (35). This is an important statement as the latter situation occurs within brain lesions, characteristic, for instance, of multiple sclerosis (36).

Inflammation, often present in diseases associated with white matter impairment, poses another difficulty for the interpretation of DTI signals (37). In a cuprizone experimental mouse model, it has been shown that axial diffusivity (λ║) values were diminished in the beginning of demyelination process in corpus callosum regions characterized by nonuniform axonal edema, beads, varicosities parallel to the axon segments, and microglia/macrophage activation. In the same animals, axial diffusivity (λ║) was not decreased during prolonged demyelination, in which axonal atrophy was evident. The radial diffusivity (λ_┴_) values generally were enhanced in chronically demyelinated corpus callosum voxels, but in regions with extensive axonal edema and prominent inflammatory cell presence, radial diffusivity (λ_┴_) did not change, likely because of reduced intra-axonal water diffusivity following injury and/or the enhanced restriction related to the presence of infiltrating cells (38).

A combination of oligodendrocyte apoptosis and the development of vasogenic edema could also result in enhanced diffusivity across the axons, leading to discrepancies between radial diffusivity (λ_┴_) and the histological picture (39). Thus, DTI-derived radial diffusivity (λ_┴_) may not be specific to myelin integrity and may actually reflect both myelin integrity and extra-axonal water content (40–42). Finally, cerebrospinal fluid contamination represents another challenge. Cheng et al. (43) proposed a combination of the DTI technique and a FLAIR *b* = 0 image to suppress cerebrospinal fluid partial volume effects and improve white matter fiber tractography.

Summing up, experimental studies from different pathogenic events: acute injury (ischemia, trauma), short term/progressive injury (cuprizone model), and congenial and long-term chronic injury (Shivered mice) are part of different cellular responses which can result in different DTI scalars anomalies (Table 1). In addition, some of the acute processes (trauma), include complex acute multicellular processes (inflammation) and chronic processes (scarring) which could radically change the white matter matrix structure and temporal course of the DTI parameters. Moreover, trophic iteration between neuronal and glial cell populations in the nervous tissue should be taken into consideration. The pathological changes occurring in one population may defectively alter another cell group and affect axial (λ_║_) and radial (λ_┴_) diffusivity.

**Table 1:** Summary of findings from specific experimental reports cited in the review.

Experimental model/disease	Axial diffusivity (λ_║_)	Radial diffusivity (λ_┴_)	Histopathological correlation	Reference
Congenitally demyelinated Shiverer mutant mice	Not changed	Increased	Yes, for axial (no axon damage—no λ_║_ change) and radial (demyelination) diffusivity	(19)

Mouse model of retinal ischemia	Decreased by day 3 after ischemia	Decreased on day 5 and present on day 7 after ischemia	Yes, at day 3 (axonal degeneration) and 7 (myelin fragmentation) after the injury	(20)

Mouse cuprizone model of experimental demyelination and myelination of corpus callosum	Tendency to decrease, but not reached statistical significance	Increased	Yes, for radial diffusivity (demyelination), only tendency for axial diffusivity (axon damage)	(29)

Mouse cuprizone model of experimental demyelination and myelination of corpus callosum	Decreased	Increased	Yes, for both axial (axon damage) and radial (demyelination)	(33)

Mathematical modeling			No, if the signal-to-noise ratio is low, if crossing fibers are present, or if pathology causes a decrease in anisotropy	(34, 35)

Rat model of liposaccharide injection into the corpus callosum		Increased	No, radial diffusivity increase due to vasogenic edema	(37)

Mouse cuprizone model of experimental demyelination and myelination of corpus callosum	Decreased	Increased	No, axial diffusivity did not correlate with axonal atrophy; did not correlate with myelin loss or astrogliosis	(38)

Mouse model of acute spinal cord injury	Increased	Increased	Good correlation in the epicenter and remotely to the changes, axial and radial diffusivity impacted by vasogenic edema	(39)

Mathematical modeling			Cellularity decrease axon diffusivity, have a limited impact on radial diffusivity; vasogenic edema increases radial diffusivity	(42)

## Clinical Aspects of Axial and Radial Diffusivity

Pathophysiological changes in multiple sclerosis encompass the dynamic evolution of inflammation, axonal injury, and myelin loss, which creates a particularly challenging situation for imaging with axial (λ_║_) and radial (λ_┴_) diffusivity. The timing of inflammation relative to tissue injury is not always known. In addition to the temporal aspect, the pathology in multiple sclerosis is also complex and variable, with axon and myelin injury strongly interlinked. Nevertheless, Oh et al. (44, 45) demonstrated that fractional anisotropy, mean diffusivity, and radial diffusivity (λ_┴_) could efficiently discriminate multiple sclerosis patients with high and low disability levels. Fractional anisotropy was diminished, mean diffusivity increased, and radial diffusivity (λ_┴_) enhanced in subjects with high disability, as compared with low disability, demonstrating good reproducibility.

Kronlage et al. (46) demonstrated that fractional anisotropy and radial diffusivity (λ_┴_) correlated strongly with electrophysiological markers of demyelination, whereas axial diffusivity (λ_║_) did not correlate with markers of axonal neuropathy in subjects with chronic inflammatory demyelinating polyneuropathy. In another study, axial diffusivity (λ_║_) and fractional anisotropy showed a significant correlation with axonal integrity, whereas radial diffusivity (λ_┴_) was related to myelin compactness in an animal model of closed head traumatic brain injury (47). In this study, fractional anisotropy was sensitive to astrogliosis in the gray matter, whereas mean diffusivity was associated with augmented cellularity.

Interestingly, Naismith et al. (48) demonstrated that in remote optic neuritis (commonly one of the first manifestations of multiple sclerosis), radial diffusivity (λ_┴_) may discriminate visual outcomes. White matter tracts consisting of parallel axons tightly packed with myelin are anisotropic, or directional, to the diffusion of water. Chronic injury associated with demyelination and axons loss leads to reduced anisotropy. As a consequence diffusion perpendicular to the white matter tract (analogous to λ_┴_) augments, overall diffusivity (mean diffusivity) increases, and tissue directionality diminishes. At the same time, within the human central nervous system, pathologic changes from the acute to the chronic stage result in axial diffusivity (λ_║_) becoming less informative over time. As myelin debris is cleared, inflammation and edema diminish, demyelinated axons are less tightly packed, and the widening interstitial space dilutes the ability of DTI to detect and measure anisotropic diffusion (λ_║_) within axons. Thus, the correlation between axial diffusivity and visual outcomes in subjects with remote optic neuritis was very modest (48).

To summarize, in cases of axon and myelin injury associated with inflammation and increased cellularity, DTI tends to underestimate the extent of demyelination, while at the same time, may exaggerate the extent of the axonal injury. The final outcome is undervalued radial diffusivity (λ_┴_) and overvalued axial diffusivity (λ_║_). In turn, in chronic diseases associated with intensive axonal loss, the increased isotropic diffusion seems to enhance both radial (λ_┴_) and axial diffusivity (λ_║_). Consequently, DTI can no longer provide sufficient reliability in terms of underlying pathologies when inflammation, axonal loss, axonal injury, and demyelination coexist.

## Future Prospects

Song and colleagues have proposed a new methodology called diffusion basis spectrum imaging (DBSI) to address the DTI inaccuracies with respect to radial (λ_┴_) and axial diffusivity (λ_║_). Phantom tests and *in vivo* experiments using cuprizone-treated mice suggest that DBSI might be capable of quantifying the extent of augmented cellularity and vasogenic edema, constituting a reliable marker of inflammation. Moreover, DBSI seems to improve the quantification of axial (λ_║_) and radial (λ_┴_) diffusivity, which distinguishes and reflects axonal versus myelin injury (40).

The DBSI model proposed by the Song research group has been validated in several animal and human studies, as reviewed by Cross and Song (49). The possible limitation of the reviewed research describing the interdependencies between axial (λ_║_) versus radial (λ_┴_) diffusivity, and axonal versus myelin injury (respectively), is that most of the discussed studies originated from one site. In particular, replication of DBSI-derived data is yet to be published. Animal models of neurodegenerative diseases featuring fluorescently labeled axons (50) represent another option to correlate axonal pathology to specific alterations in axial and radial diffusivities. Further development of DTI technology, including DBSI and other concepts (such as specific animal models), may enormously advance our understanding of underlying pathologies in several central nervous disorders.

Diffusion tensor imaging can reveal strategic information with respect to white matter tracts, disconnection mechanisms, and related symptoms. Axial (λ_║_) and radial (λ_┴_) diffusivity seem to provide quite consistent information in healthy subjects, and in pathological conditions with limited edema and inflammatory changes. DTI remains one of the most promising non-invasive diagnostic tools in medicine.

## Author Contributions

PW and AS drafted and wrote the manuscript. PN, KJ, ES, and AS commented and revised the manuscript. All authors have read and approved the final version of the manuscript.

## Conflict of Interest Statement

The authors declare that the research was conducted in the absence of any commercial or financial relationships that could be construed as a potential conflict of interest.
